# Ophiopogonin B inhibits migration and invasion in non-small cell lung cancer cells through enhancing the interaction between Axin and β-catenin

**DOI:** 10.7150/jca.60066

**Published:** 2021-08-28

**Authors:** Shiping Zhang, Hongxiao Li, Liqiu Li, Qian Gao, Ling Gu, Cheng Hu, Meijuan Chen, Xu Zhang

**Affiliations:** 1 School of Medicine &Holistic Integrative Medicine, Nanjing University of Chinese Medicine, Nanjing, 210023, P.R. China; 2 Health center, Nanjing University of Chinese Medicine, Nanjing, 210023, P.R. China

**Keywords:** non-small cell lung cancer cells (NSCLC), migration, invasion, Wnt/β-catenin pathway, EMT

## Abstract

Ophiopogonin B (OP-B), a kind of saponin compound that exists in *Radix Ophiopogonis* is frequently adopted for the treatment of lung disease as traditional Chinese medicine. The present work aimed to explore the anti-tumor activity of OP-B on non-small cell lung carcinoma (NSCLC) and its possible mechanism. We found that OP-B-treated cells suppressed the viability and proliferation of cells depending on its concentration, as assayed by MTT and Alamar Blue (IC_50_ were 14.22 ± 1.94, 12.14 ± 2.01, and 16.11 ± 1.83 μM in A549, NCI-H1299, and NCI-H460 cells, respectively). Then, the suppressive effect of OP-B on the invasion and migration of NSCLC was observed through wound healing and Transwell assays, and the epithelial-mesenchymal transition (EMT) markers was detected by immunofluorescence and western blotting. In addition, a dose-dependent reduction of β-catenin both within cytoplasm and nucleus was observed, and the downstream proteins cyclin D1 and c-Myc of Wnt/β-catenin pathway were also reduced. We further constructed β-catenin-overexpression cell models to reveal the underlying mechanism. The results showed that 10 μM of OP-B notably reduced β-catenin protein levels, as well as cell migration and invasion. In spite of the increasement of β-catenin, activation of Wnt pathway and EMT progression, knockdown of Axin leaded to de-function of OP-B on cell metastasis. Taken together, OP-B reduced NSCLC migration and invasion by strengthening the Axin/β-catenin interaction and reducing β-catenin protein translocation.

## Introduction

Lung cancer (LC) accounted for the primary reason for cancer incidence and death in the world 1, which was responsible for 11.6% of the cancer cases in 2018 2. Non-small cell lung carcinoma (NSCLC) took up about 85% LC cases, and a majority of patients had low recovery rates due to limited conventional therapies, tumor metastasis, and recurrence 3. Hence, it is vital to seek out better therapeutic methods or anti-cancer agents with less toxic side effects.

Mounting evidence indicated that bioactive natural compounds from traditional medicine could be used as alternative anti-cancer agents. Saponins were abundant compounds in medicinal plants. Due to their triterpene or steroid aglycone structure, saponins behaved in a wide range of biological properties, including anti-cancer, anti-oxidation, anti-inflammation, and anti-virus 4-5. It had been reported that saponins functioned as potent inhibitors on tumor growth by inducing apoptosis, stabilizing microtubules, and deregulating several signal transduction pathways 6. For example, the combination of Paris saponin Ⅱ and curcumin caused LC cell apoptosis through suppressing PI3K/Akt, PCNA, and NF-κB pathways, as well as activating several death receptors, including CD40/CD40L, DR6, TNF-α and FasL 7. It was also reported that apoptosis-induction in cancer cells were associated with ROS production 8. Recently, emerging studies showed that saponins had broad-spectrum anti-tumor activity including their anti-invasion properties 9.

Metastatic dissemination was believed to be one of the main tumor processes to evade traditional therapies, contributing to 90% of death worldwide. Epithelial-mesenchymal transition (EMT) was characterized by the loss of cell-cell interactions, apicobasal polarity, and gain of mesenchymal properties. Executed by various epithelial carcinomas, EMT was found to initiate cell invasion and metastasis during tumor development. The decrease of E-cadherin expression while maintaining the integrity of basal membrane as well as the differentiation of epithelial phenotype, were hallmarks of epithelial cells undergoing EMT. Some EMT regulators, like snail and slug suppressed this process, while up-regulation of vimentin accelerated EMT process, resulting in the dismal patient survival 11. Wnt/β-catenin signaling was considered to be the crucial signaling pathway in LC cell proliferation, survival, and metastasis 12,13. The central transcription factor, β-catenin, aberrantly accumulated within cytoplasm, leading to β-catenin stabilization and nuclear translocations. There β-catenin bound to the transcription factors T-cell factor (TCF)/lymphoid enhancer factor (LEF) and activated the downstream target proteins, including c-Myc and cyclin D1^14^.

More studies suggested that herbal medicines showed promise as lung cancer therapeutics with less toxicity and clinical advantages. *Radix Ophiopogonins*, one of the traditional Chinese herbs, was widely used as a common component of classic lung cancer prescriptions in clinical settings, such as *Mai Men Dong Decoction* and *Ke Jinyan Prescription*. Ophiopogonin B (OP-B) was the saponin extracted from *Radix Ophiopogon japonicus*, which possessed cell cytotoxic activity against 11 lung cancer cell lines. Recent studies revealed that OP-B could induce autophagy, mitotic catastrophe and apoptosis of H157 and H460 cells by inhibiting PI3K/Akt signal transduction pathway 15. It also exerted an important part in suppressing adenocarcinoma cell metastasis and angiogenesis *in vitro* and *in vivo* 16. However, a full understanding of the molecular mechanisms behind OP-B's anti-invasion effects was still unclear.

The present work discovered that OP-B inhibited NSCLC cell line invasion and migration. Considering EMT was a pathological event related to the metastatic cascade with tumor progression and invasion, we proposed that the OP-B inhibitory effect on NSCLC would be associated with EMT. Since EMT could be regulated by the Wnt signaling pathway 17, therefore, this study was carried out to explore whether Wnt pathway was involved in the OP-B's regulatory effect on NSCLC cells.

## Materials and methods

### Reagents and antibodies

Ophiopogonin B (MW: 722.90238 g/mol, purity ≥ 98%) was provided by Nanjing Ze Lang Medical Technology Co., Ltd (Jiangsu, China). The stock was prepared by dissolving the compound in dimethyl sulfoxide (DMSO, Sigma, US). The stock was frozen at -20°C. Culture medium was used to dilute OP-B to suitable doses before use, while the DMSO final concentration was less than 0.01%. Primary antibodies anti-β-catenin (8480), anti-phospho-β-catenin (Ser33/37/Thr4) (9561), anti-cyclin D1 (2978), anti-c-Myc (5605), anti-E-cadherin (14472), anti-vimentin (5741), anti-snail (3879), anti-slug (9585) and anti-LainB1 (13435) were provided by Cell Signaling Technology. Meanwhile, anti-β-actin antibody (sc4778) was provided by Santa Cruz Biotechnology. Anti-Axin1 (ab233652) and anti-GSK3β (ab93926) antibodies were purchased from Abcam. Goat anti-rabbit and anti-mouse horseradish peroxidase (HRP)-conjugated secondary antibodies were provided by Cell Signaling Technology.

### Cell lines and culture

A549 and NCI-H460 cells were obtained from KeyGEN Bio TECH Corp., Ltd (Jiangsu, China). NCI-H1299 cells were obtained from Procell Life Science and Technology Co., Ltd (Hubei, China). A549 cells were cultivated within DMEM and F12 medium (Gibco, US), while NCI-H460 and NCI-H1299 cells were cultured within the RPMI 1640 medium (Gibco, US). 10% fetal bovine serum (FBS, Gibco, USA) and 100 U/ml penicillin-streptomycin mixed antibiotics were added into each medium. Each cell line was kept under 37°C in a humidified 5% CO_2_ atmosphere.

### Lentivirus construct and transfection

The overexpression β-catenin lentivirus (LV-CTNNB1) (OE), negative control lentivirus (NC), shRNA Axin1 lentivirus (LV-AXIN1-RNAi) (KD), and sh-negative control lentivirus (sh-NC) were obtained from KeyGEN Bio TECH Corp., Ltd (Jiangsu, China). Cells were seeded into the 12-well plate when reaching 50% confluence, followed by lentivirus transfection. The containing lentivirus medium was removed after 12 h. Subsequent experiments were carried out 48 h after transfection.

### Cell viability assay

Cells (5×10^3^/well) were inoculated into the 96-well plates with 100 μl suspension in each well. One day before experimental treatment, OP-B at different doses were used to treat cells. The Cell Counting Kit-8 (CCK-8, Dojindo, Japan) assay and Alamar Blue kit (Thermo Fisher, US) assay were used to analyze cell viability. After culturing cells with OP-B incubation, cells were added with 10 μl CCK-8 solution or 10 μl Alamar Blue solution for another 30 min of incubation at 37°C. The microplate reader was utilized to measure absorbance for every treatment at wavelengths 450 nm or 570 nm.

### Wound-healing assay

A549 and NCI-H1299 cell lines were seeded into the 6-well plates at 4 × 10^4^ cells/well. At approximately 48 h following incubation when cells achieved 100% confluence, the sterile pipette tip (200 μl) was adopted for making a scratch in the middle of every well. Then, all wells were rinsed by phosphate buffer saline (PBS) to remove cell debris and those non-adhering cells. Then, cells were incubated with OP-B to various doses or transfected with lentivirus for 24 h incubation. Wound edge movement images were obtained with a microscope. The distance migrated was calculated by dividing the distance at the time point by the distance at the beginning (Migration rate= (distance at 0h-distance at 24h) ×100%/distance at 0h). For each experiment, a total of 5 wounds were measured per group, and the results were analyzed using Image J Software.

### Cell invasion assay

Cell invasion assay was carried out by the 0.8 μm permeable transwells (Corning, US). Matrigel (Corning, US) was used to cover the upper chamber, followed by the addition of 10% FBS media. Media with 20% FBS, which served as the chemoattractant, was added into the lower chamber. After incubation at 37°C overnight, serum-starved cells (2 × 10^5^) were exposed to OP-B treatment at different doses or transfected with lentivirus within upper transwell chambers for 24 h. Then, the cotton swab was used to remove non-invading cells in the upper chamber. Upper chambers were then subjected to 30 min of 4% paraformaldehyde fixation and 1% crystal violet staining. The number of invasive cells was quantified by counting the number of cells in five randomly chosen fields at 200× magnification under the microscope (Leica, Germany). (Invasive rate=invasive cells in OP-B groups ×100%/invasive cells in control groups).

### Immunofluorescent staining

The A549, NCI-H1299, and NCI-H460 cell lines (5 × 10^4^/well) were inoculated into the 24-well plates to incubate until reaching 60% confluence. The cells were subsequently treated with 10 μM OP-B. After 24 h of incubation and cold PBS washing twice, cells were subjected to 30 min of 4% paraformaldehyde fixation under ambient temperature. Afterwards, each well was added with 500 μl 0.2% Triton X-100 for 10 min of cell permeabilization. After incubation 60 min with 1% BSA to block the nonspecific binding, primary antibodies against E-cadherin or vimentin (1:100) were used for overnight immunolabeling under 4°C. On the next day, FITC-conjugated goat anti-rabbit IgG secondary antibody (1:250) was added to incubate cells for 2 h under ambient temperature. DNA was visualized 10 min after the addition of DAPI. The fluorescence microscopy (OLYMPUS, Japan) was employed in obtaining images.

### Western blot assay

The A549, NCI-H1299, and NCI-H460 cell lines were collected at 24 h following treatment or transfection and lysed in a protein extraction buffer for 30 min at 4°C. The Nuclear and Cytoplasmic Protein Extraction Kit (Beyotime Biotechnology) was utilized to extract nuclear protein. The lysate concentrations were adjusted by measuring protein content according to bicinchoninic acid (BCA) method. Altogether 50 μg protein blended with 5× SDS loading buffer was added to 10%-15% SDS-PAGE gels. After first dimension separation, the protein was then transported onto the PVDF membranes. Later, 5% non-fat milk was used to block the membranes under ambient temperature and subsequently incubated using those above described primary antibodies under 4°C overnight, followed by 2 h of incubation using the HRP-labeled anti-mouse (1:500) or anti-rabbit (1:500) secondary antibody under room temperature. Later, the Gel DocTM XR^+^ Gel Documentation System (Bio-Rad, US) was utilized to detect bands using the enhanced chemiluminescence (ECL) reagents.

### Co-immunoprecipitation (Co-IP) assay

After washing with cold PBS twice, 3 ml co-IP cell lysis buffer (1% Triton X-100, 150 mM NaCl_2_, 1.5 mM MgCl_2_, 50 mM HEPES pH 7.6, 1 mM EDTA, 10% glycerol, 10 mM NaF, 1 mM NaVO_3_, 10 mM β-glycerolphosphate, 50 ml DDM, and 5 protease inhibitor tablets) was placed into each cell scraped a 10 cm dish. Whole protein samples were obtained by vortexing and centrifuging the debris. 14 μl of anti-β-catenin or 7 μl of anti-Flag antibodies were added to every 1.5 ml microcentrifuge tube containing 600 mg protein within 700 μl co-IP buffer. After incubation overnight at 4 °C, the cell lysate was subjected to 4 h of incubation with 20 μl protein-G sepharose under 4°C with rocking. Then 500 μl RIPA buffer was used to wash beads for 4 times, followed by boiling in SDS loading buffer. 80 μl whole cell lysates were boiled with SDS loading buffer as a control for input. The following gel and blotting procedures were the same as the western blotting assay.

### Statistical analysis

The statistical significance of the experimental data was analyzed by SPSS 21.0 software. The data were expressed as the mean ± standard deviation (SD). Differences between three or more groups were analyzed using one-way ANOVA and least-significant difference (LSD). All statistical tests were two sided. *P* < 0.05 was considered statistically significant. Figures were produced using GraphPad Prism 5 and Adobe Illustrator.

## Results

### OP-B inhibited NSCLC proliferation, motility, and invasiveness

**Fig 1:**
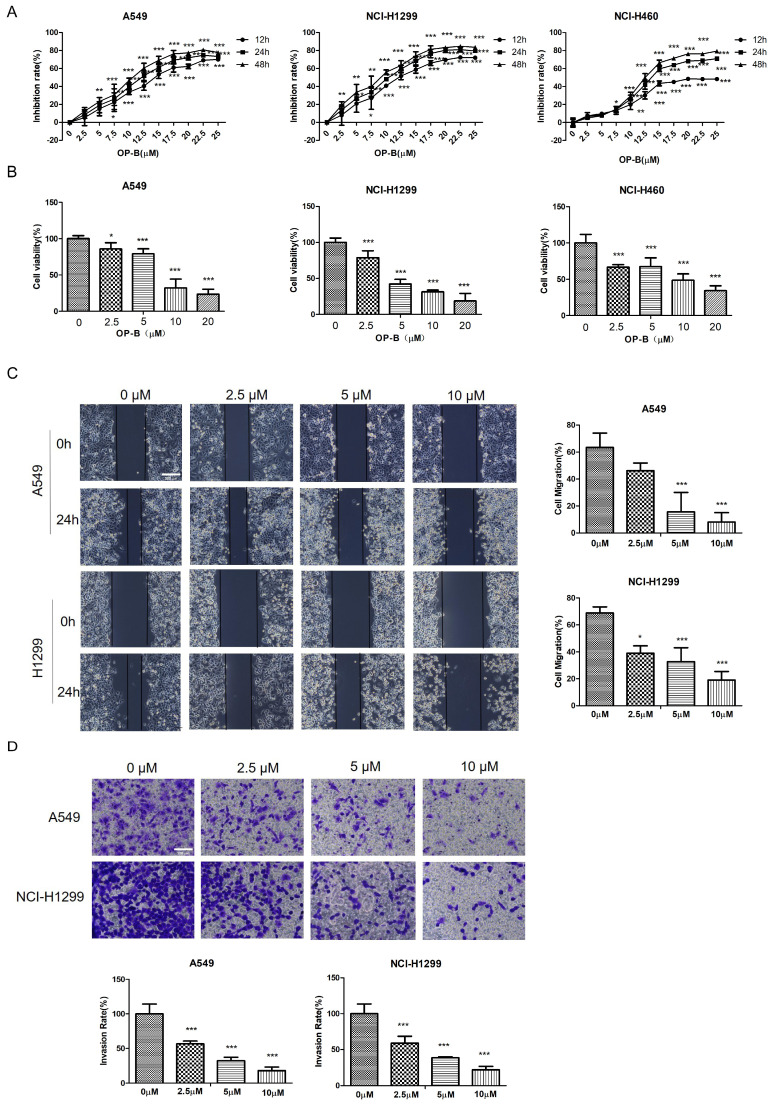
OP-B reduces the proliferation, migration and invasion of NSCLC cells. A. CCK-8 proliferation assay of A549, NCI-H1299 and NCI-H460 cells treated with different concentrations of OP-B for 12 h, 24 h or 48 h. B. Alamar blue viability assay of A549, NCI-H1299 and NCI-H460 cells treated with OP-B for 24 h. C. Wound healing assay of A549 and NCI-H1299 cells treated with OP-B for 24 h. Scale bar: 200 µm. D. Transwell invasion assay of A549 and NCI-H1299 cells treated with OP-B for 24 h.Scale bar: 100 µm. The bars and error bars indicate the mean ± SD. All the data are analyzed using one-way ANOVA and least-significant difference (LSD). *P < 0.05, **P < 0.01, ***P < 0.001.

We first examined whether OP-B affected NSCLC growth by CCK8 assay and Alarm Blue assay. OP-B exerted a significant inhibitory effect on the proliferation of A549, NCI-H1299, and NCI-H460 cell lines depending on the time and concentration. Typically, IC_50_ values were 14.22 ± 1.94, 12.14 ± 2.01, and 6.11 ± 1.83 μM respectively (Fig. 1A). Compared to the control group, the viability rates of NSCLC cells with 20 μM OP-B treatment decreased more than 30% (Fig. 1B).

To further clarify the OP-B anti-cancer effect, the migratory potential of A549 and NCI-H1299 cell lines were tested using the wound-healing assay under OP-B incubation at 24 h. As in Fig. 1C, the wound areas were larger in cells which were treated with higher concentrations of OP-B, suggesting OP-B inhibited cells migratory activity. Similar to the wound-healing results, OP-B also decreased the invasive capacity of A549 and NCI-H1299 cell lines. Relative to control group, invasion rates decreased to nearly 50% in OP-B (2.5 μM) treatment group (Fig. 1D). Together, these findings indicated that OP-B suppressed cell growth, migration, and invasion in NSCLC.

### OP-B reversed EMT and suppressed β-catenin transcriptional activity

**Fig 2:**
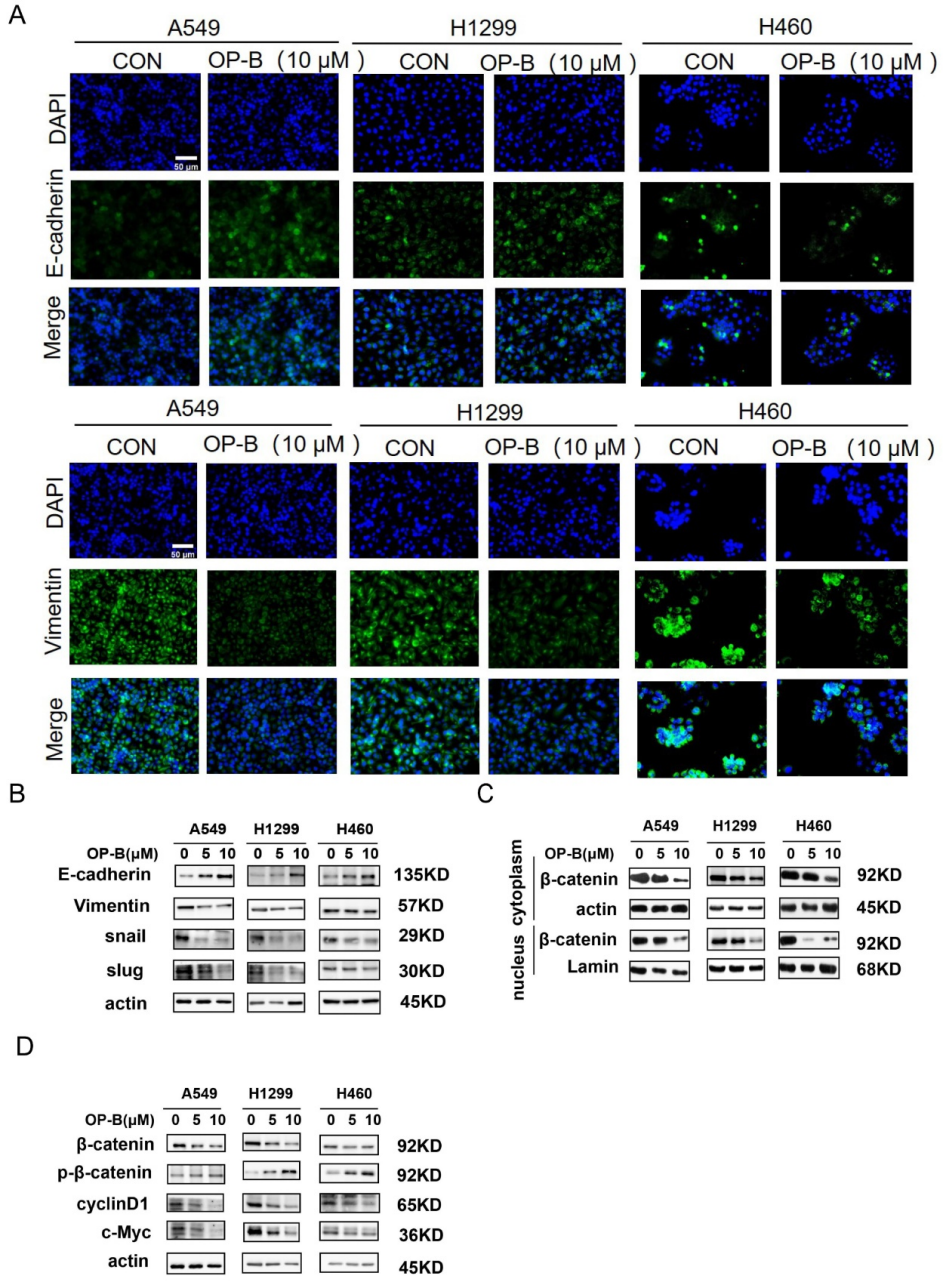
OP-B suppresses the EMT progression and Wnt/β-catenin signaling pathway. A. The affection of OP-B on the immunofluorescence density of EMT markers in A549, NCI-H1299 and NCI-H460 cells. Scale bar: 50 µm. B. The immunoblotting expression of EMT markers in NSCLC cells treated with OP-B. C. The immunoblotting expression of β-catenin protein levels in the cytoplasm and nucleus on A549, NCI-H1299 and NCI-H460 cells treated with OP-B. D. The affection of OP-B on the expression of β-catenin, p-β-catenin, cyclinD1 and c-myc in A549, NCI-H1299 and NCI-H460 cells.

It was known that EMT was strongly associated with carcinoma invasion and metastasis. However, the effects of OP-B on EMT progression remained unclear. Since the above experiments suggesting IC_50_ of OP-B on A549, NCI-H1299, and NCI-H460 cell lines were 14.22 ± 1.94, 12.14 ± 2.01, and 6.11 ± 1.83 μM, we chose 10 μM OP-B as candidate concentration to evaluate OP-B effect on NSCLC cells. The results of immunofluorescence staining indicated the EMT-related markers were regulated, E-cadherin level increasing and vimentin decreasing in A549 and NCI-H1299 cell lines, while no obvious change of immunofluorescence staining intense was observed in NCI-H460 cells (Fig. 2A). The results of western blots were also identical to the immunofluorescence assay. Exposing NSCLC to various concentrations of OP-B (0 to 10 μM) led to rising E-cadherin protein levels, and vimentin protein levels dramatically declined. Moreover, OP-B-cells exhibited significantly decreasing snail and slug expression, which were E-cadherin's transcriptional repressors (Fig. 2B).

Mounting evidences suggested that the Wnt/β-catenin signaling pathway was the positive regulator for cancer cell proliferation, survival, and metastasis. To better understand whether OP-B modulated the Wnt/β-catenin signal transduction pathway in NSCLC, this study monitored β-catenin protein levels after OP-B treatment. Western blot results showed that OP-B stimulated β-catenin phosphorylation depending on its dose. Consistent with this observation, OP-B also adversely affected β-catenin transcription activity and nuclear translocation (Fig. 2C), as observed by the down-regulated protein levels of cyclin D1 and c-Myc (Fig. 2D).

### OP-B reversed EMT to enhance NSCLC invasion induced by β-catenin

**Fig 3:**
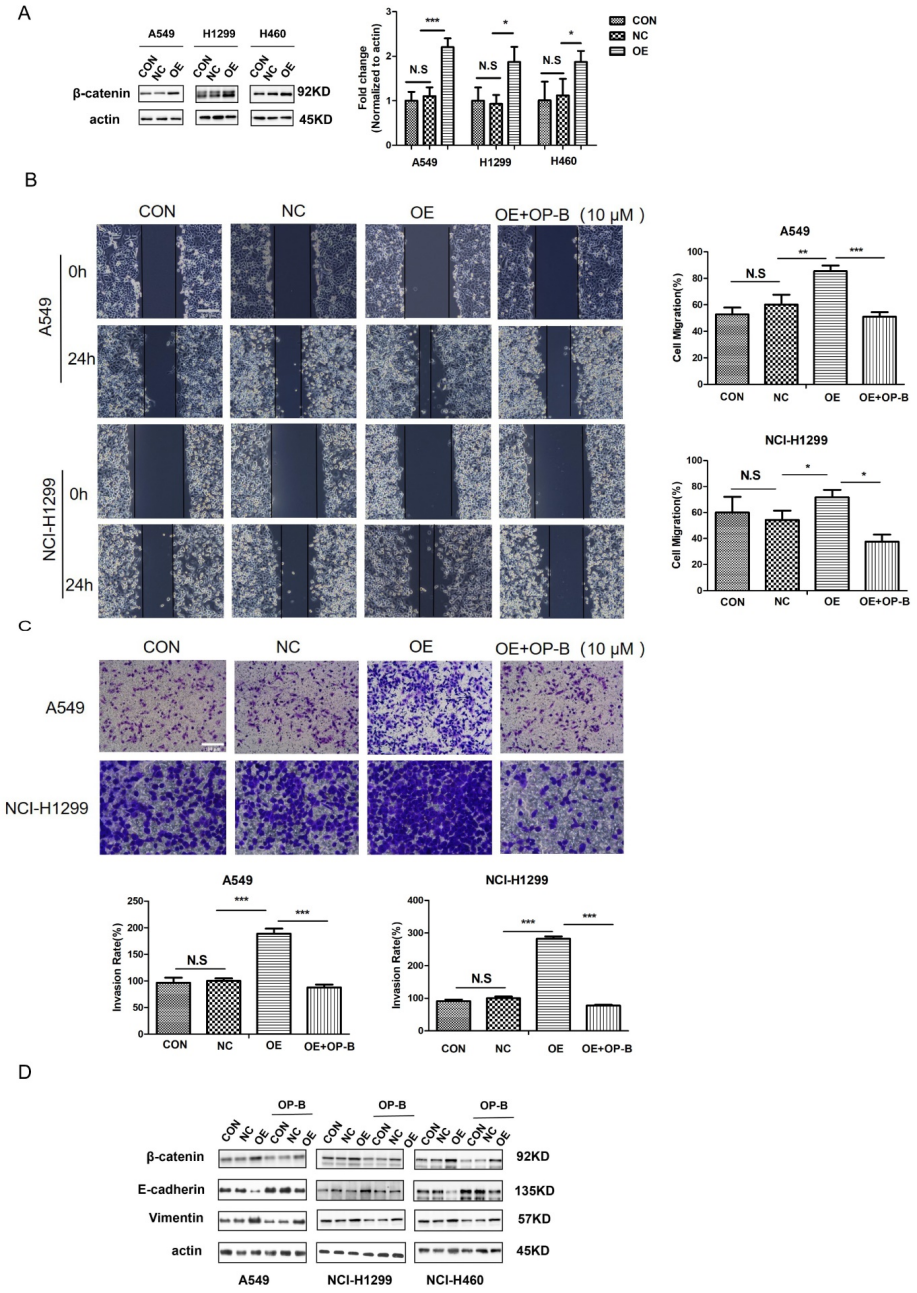
OP-B suppresses the migration and invasion of NSCLC cells by reversing EMT and inhibiting β-catenin expression. A. The validation of β-catenin over expression by LV-CTNNB1 in A549, NCI-1299 and NCI-H460 cells determined by western blot. B. Regulation of OP-B on cells migration in β-catenin-overexpressed A549 and NCI-1299 cells detected by wound healing assay. Scale bar: 200 µm. C. Regulation of OP-B on cells invasion in β-catenin-overexpressed A549 and NCI-1299 cells detected by Transwell assay. Scale bar: 100 µm. D. Detection of EMT signaling pathway and β-catenin in A549 and NCI-1299 cells transfected by LV-CTNNB1 within or without OP-B incubation. The bars and error bars indicate the mean ± SD. All the data were analyzed using one-way ANOVA and least-significant difference (LSD). *P < 0.05, **P < 0.01, ***P < 0.001.

Since free β-catenin was known as a canonical Wnt signaling transcriptional co-activator and contributed to cell tumorigenesis, we aimed to explore whether β-catenin was involved in OP-B functional effect on cell motility. The western blot results showed that the β-catenin protein levels in NSCLC cells transfected with LV-CTNNB1 were much higher than those in control groups or cells transfected with negative control lentivirus (Fig. 3A). As Fig. 3B illustrated that β-catenin stimulation produced significantly increased migration rates, which were enhanced 85.44% ± 4.17% and 71.67% ± 5.77% in A549 and NCI-H1299 cells, respectively. By contrast, β-catenin overexpression cells incubated with 10 μM OP-B exhibited a dramatic decrease in migration, the migration rates of which decreased to 51.75% ± 3.36% and 37.50% ± 5.51%.

Furtherly, the results of transwell assay indicated that OP-B could dramatically inhibit the cell invasions which were induced by overexpressed β-catenin. Cell invasive rates decreased to 87.88% and 77.46% in response to OP-B treatment under β-catenin overexpression, whereas 188.89% and 282.85% invasion rates were observed in OE groups (Fig. 3C).

Cancer cells undergoing EMT was wildly accepted to be the early event resulting in local invasion together with distant metastasis, therefore, in order to determine the association of cell metastasis with OP-B-mediated EMT conversion, we tested β-catenin and EMT markers expression. Compared to the control group, we observed a remarkable reduction in vimentin levels and a distinct increase of E-cadherin expression in β-catenin overexpression cells. While, OP-B (10 μM, 24h) treatment induced a suppressive effect on β-catenin, and reversed EMT in β-catenin overexpression cells (Fig. 3D). These observations suggested that β-catenin, regulating EMT conversion, could be suppressed by OP-B. Taken together, the data above showed that the downregulation of aggressive invasion by OP-B was tightly related to β-catenin inhibition and EMT reversion.

### Axin1 interacted with β-catenin and played a critical role in OP-B downregulating β-catenin

**Fig 4:**
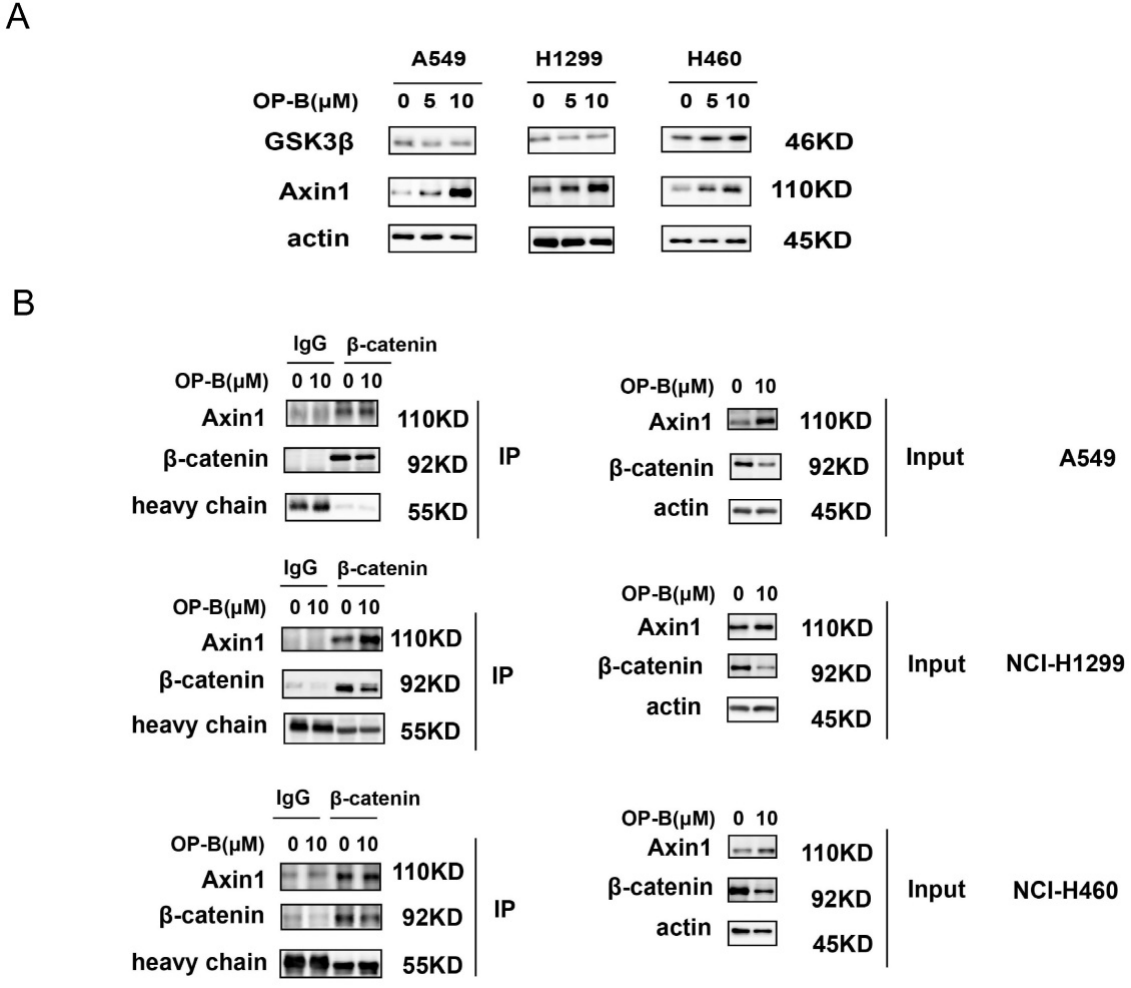
The positive role of OP-B on the interaction between Axin and β-catenin. A. The expression of GSK3β and Axin1 in A549, NCI-H1299 and NCI-H460 cells treated with OP-B. B. Effect of OP-B on the binding of Axin1 to β-catenin in A549, NCI-H1299 and NCI-H460 cells were detected by co-IP.

The β-catenin content in cytoplasm was believed to be modulated through the destruction complex that contained glycogen synthase kinase 3β (GSK3β) and Axin. Therefore, we assessed whether OP-B mediated β-catenin level through the destruction complex. As suggested by western blotting, GSK3β protein expressions were not consistent in A549, NCI-H1299, and NCI-H460 cells, while, Axin1 protein levels were concurrently enhanced in OP-B treated cells (Fig. 4A). To further validate OP-B influence on the interaction of Axin1 and β-catenin, we performed co-IP. As Fig. 4B showed that β-catenin protein levels decreased after OP-B incubation both in input and IP samples, but 10μM OP-B treatment caused the Axin levels in IP samples to increase or be equal, suggesting OP-B facilitated the binding between Axin to β-catenin.

### OP-B inhibited NSCLC motility and metastasis by enhancing Axin stabilization

**Fig 5:**
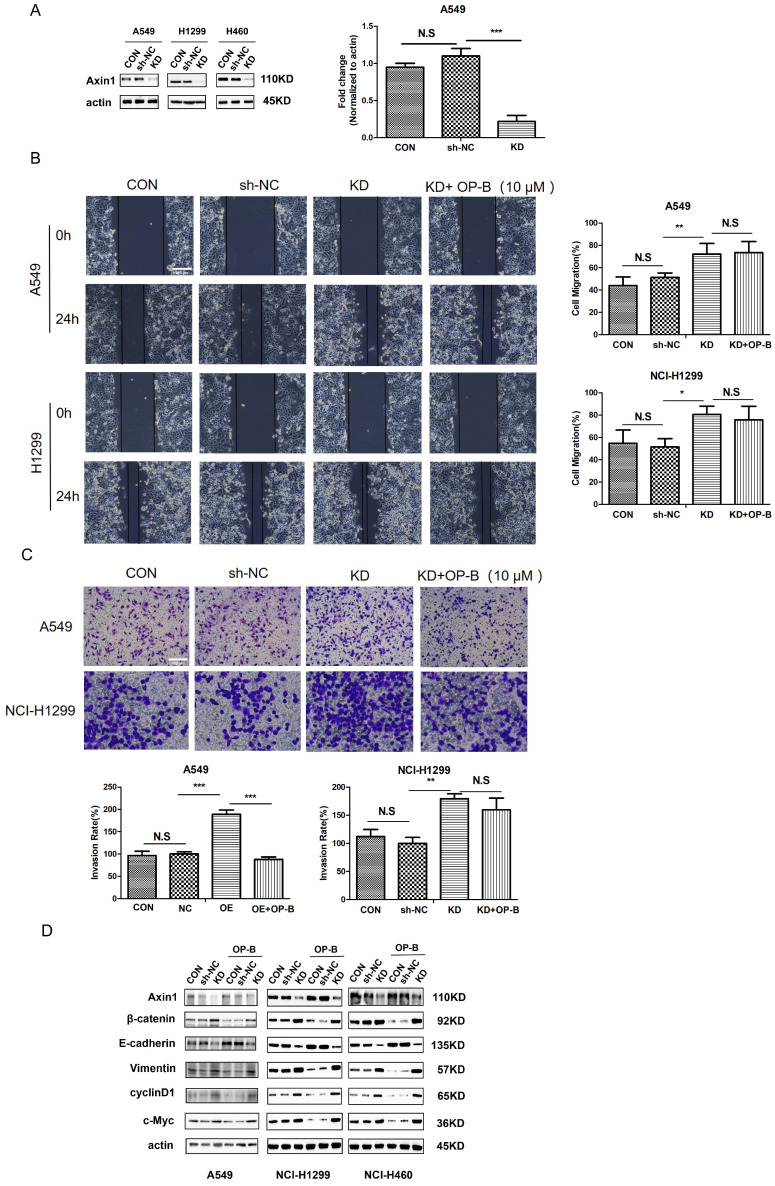
Axin is essential for the EMT reversion, Wnt/β-catenin downregulation and inhibition of cells migration /invasion which is induced by OP-B. A. The validation of Axin knockdown by LV-AXIN1-RNAi in A549, NCI-1299 and NCI-H460 cells determined by western blot. B. Regulation of OP-B on cells migration in sh-Axin A549 and NCI-1299 cells detected by wound healing assay. Scale bar: 200 µm. C. Regulation of OP-B on cells invasionin sh-Axin A549 and NCI-1299 cells detected by Transwell assay. Scale bar: 100 µm. D. Detection of Wnt/β-catenin pathway signal and EMT markers in NSCLC cells after LV-AXIN1-RNAi transfection within or without OP-B incubation (10 μM, 24h). The bars and error bars indicate the mean ± SD. All the data were analyzed using one-way ANOVA and least-significant difference (LSD). *P < 0.05, **P < 0.01, ***P < 0.001.

To address the function of Axin in NSCLC, we first employed shRNA Axin1 lentivirus to silence Axin expression (Fig. 5A). Then we performed wound-healing and Transwell assay to ascertain whether Axin1 was involved in the anti-cancer effect of OP-B on cell migration. As shown in Fig. 5B and 5C, silencing Axin expression enhanced the cells migration and invasion activity. However, OP-B did not significantly reduce cell migration and invasion while Axin was knockdown. That suggested that Axin was necessary for OP-B-mediated anti-migration and anti-invasion activity in NSCLC.

Considering that OP-B promoted Axin1 interaction with β-catenin protein, we transfected cells with LV-AXIN1-RNAi to verify whether this binding contributed to anti-migration and anti-invasion mechanism of OP-B. As Fig. 5D demonstrated, compared with a scrambled siRNA control, Axin1 knockdown elevated the level of vimentin and reduced expression of E-cadherin, suggesting EMT progressed. Then we sought to elucidate whether the transition of EMT was accompanied by Wnt/β-catenin pathway activation in AxinRNAi cells. The results of western blot showed that Axin1 knockdown increased the levels of β-catenin and the downstream proteins, cyclin D1 and c-Myc. Interestingly, OP-B had no obvious effect on EMT markers and the Wnt/β-catenin signal transduction pathway within Axin1 knockdown cells. Collectively, these data indicated that Axin contributed to the OP-B anti-metastasis effect in NSCLC.

## Discussion

Because of their beneficial activities, natural compounds had recently become an area of extensive research for their anti-tumor mechanism 18. Studies showed that nearly 74.9% of approved anti-cancer treatment agents were rooted in natural compounds 19. OP-B was an active natural compound found in the Chinese plants, like *Radix Ophiopogonins,* which had functional inhibitory effects on NSCLC lines. In this work, OP-B was found to suppress the proliferation, invasion and migration of A549 and NCI-H1299 cell lines.

EMT, recognized as a part of classical metastasis progression 20, was associated with poor prognosis because it was involved in many tumor characteristics, such as tumor occurrence, metastasis, invasion and treatment resistance 21. Thus, reversing EMT process may be a promising strategy to prevent cancer metastasis. EMT was also frequently investigated in natural compound's anti-cancer effect 22. In addition, loss of E-cadherin was reported to be associated with β-catenin nucleus accumulation 23, 24. β-catenin was a key molecule of conventional Wnt/β-catenin pathway which was aberrantly activated in many human cancers 25, 27, accompanied by the increasing tumor invasion activity. Wnt binding to their receptors on the cell surface initiated the signaling pathway, dissociating β-catenin destruction complex, hindering phosphorylation and stabilizing β-catenin. Then, the stabilized β-catenin entered nucleus, later interacted with TCF/LEF to turn on downstream target genes. Therefore, we firstly detected the β-catenin levels in cytoplasm and nucleus, the results of which indicated that OP-B attenuated β-catenin expression both in cytoplasmic and nucleus dose dependently by controlling phosphorylation event (Fig. 2C and Fig. 2D), then reversing EMT and preventing NSCLC invasion. The EMT reversion was characterized by a dose-dependent elevation of E-cadherin whereas a decline in vimentin protein, and snail/slug downregulation (Fig. 2B). Thus, we concluded that OP-B reversed EMT conversion on NSCLC cells through suppress β-catenin accumulation.

Then, we overexpressed β-catenin in NSCLC cells by lenti-virus transduction to explore how β-catenin acted on metastasis inhibition of OP-B. The results showed that β-catenin overexpression promoted EMT conversion, cell migration and invasion, which were all significantly suppressed by OP-B. Thus, we believed that OP-B inhibited NSCLC cells metastasis by down-regulating β-catenin level and EMT conversion. As the level of β-catenin relied on multiple proteins, we then focused on the scaffold and kinase proteins, Axin and GSK3β. Western blot results clarified that Axin levels were efficiently elevated in OP-B-treated cells compared to the control cells, while GSK3β expressions were not consistent in three NSCLC cell lines. Thus, Axin was supposed to facilitate OP-B to elevate the phosphorylation and subsequent degradation of β-catenin, and Co-IP results confirmed that OP-B promoted Axin binding to β-catenin.

Axin directly interacted with β-catenin, GSK3β, and adenomatous polyposis coli in the destruction complex. Recent studies of Axin focused on its function to β-catenin. Lei Ji *et al*. showed that USP7 inhibited Wnt/β-catenin by interacting with Axin TRAF domains and promoting the de-ubiquitination and stabilization of Axin 28. It was also reported that Axin stability can be maintained by a small molecule, XAV939, to antagonize Wnt signaling through the inhibition of tankyrase 1 and tankyrase 2, the poly-ADP-ribosylating enzymes 29. Although Axin mechanisms in the Wnt/β-catenin signal transduction pathway had been determined, the specific Axin function in OP-B-mediated Wnt pathway regulation and NSCLC invasion was not fully understood. To further ascertain the biological Axin function in OP-B treated cells, we constructed an Axin knockdown model. The following results showed that Axin-RNAi induced metastasis in NSCLC, which was characterized by EMT process. Moreover, Axin knockdown induced β-catenin accumulation, resulting in downstream proteins activation (*cyclin D1* and *c-Myc*). Interestingly, OP-B could no longer play its inhibitory effect on Wnt/β-catenin signaling, EMT conservation and cell migration/invasion in Axin knockdown cells. This observation suggested that Axin was necessary for OP-B-inhibited cell migration and invasion.

In conclusion, our study demonstrated that OP-B strongly suppressed metastatic behaviors in A549 and NCI-H1299 cells via EMT reversion, which needed Axin to target β-catenin. Our present study may provide insight into the underlying mechanism involved in OP-B anti-tumor effect on NSCLC, supporting the OP-B use in a clinical setting.
