# Ophiopogonin B inhibits migration and invasion in non-small cell lung cancer cells through enhancing the interaction between Axin and β-catenin: Erratum

**DOI:** 10.7150/jca.98623

**Published:** 2024-05-30

**Authors:** Shiping Zhang, Hongxiao Li, Liqiu Li, Qian Gao, Ling Gu, Cheng Hu, Meijuan Chen, Xu Zhang

**Affiliations:** 1 School of Medicine &Holistic Integrative Medicine, Nanjing University of Chinese Medicine, Nanjing, 210023, P.R. China; 2 Health center, Nanjing University of Chinese Medicine, Nanjing, 210023, P.R. China

Due to our carelessness and confused data saving management, we misplaced the images of trans-well, wound-healing and western-blot in the published article.

1. The western blot images of slug for H1299 cell in Fig.2B and c-Myc for A549 cell in Fig.2D were misplaced. We put the right pictures to correct the errors.
2. The wound-healing image of control group 24h for H1299 cells (Fig.3B) was misplaced. The trans-well image of NC group for H1299 cells in Fig.3C was misused. The western blot images of β-catenin, actin for H460 cells and vimentin for H1299 cells in Fig.3D were misplaced. We put the right pictures to correct these errors. The corresponding statistical charts are also updated.
3. The wound-healing images of KD+OP-B group (0h) and sh-NC group (24h) for H1299 cells were misplaced in Fig.5B. The trans-well image of sh-NC group for A549 cells in Fig.5C was misused. We put the right images in Fig.5 and update the corresponding statistical charts.

The authors apologize for the inadvertent mistakes in the initially published version and state the correction does not change the overall conclusion.

## Figures and Tables

**Fig 2:**
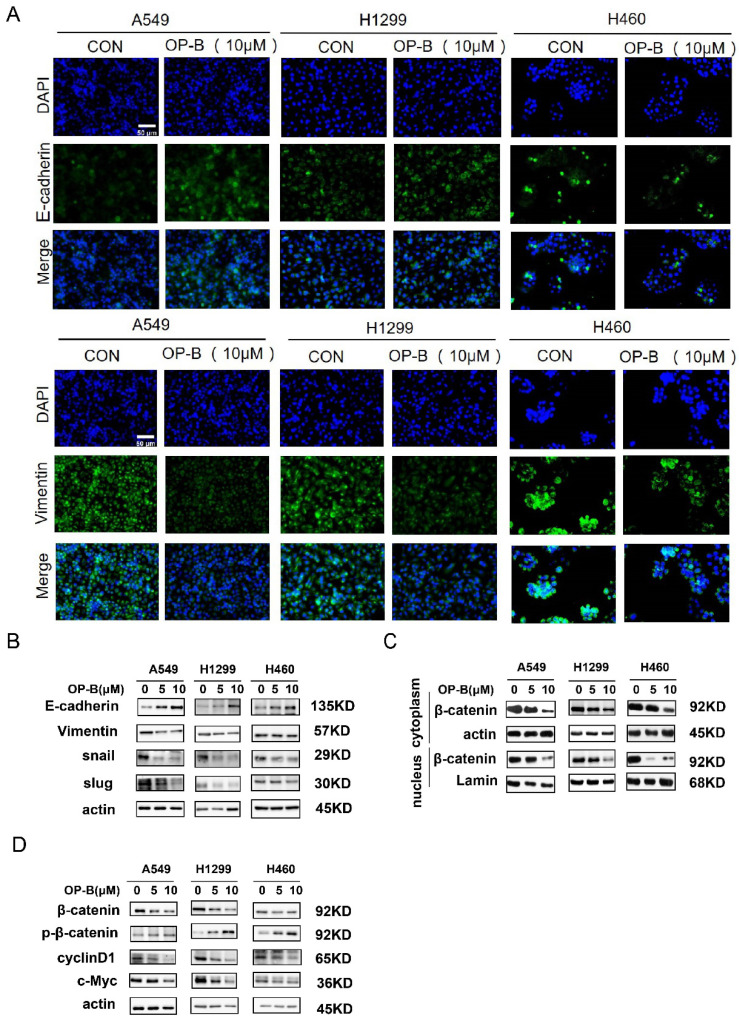
The correct new figure is shown.

**Fig 3:**
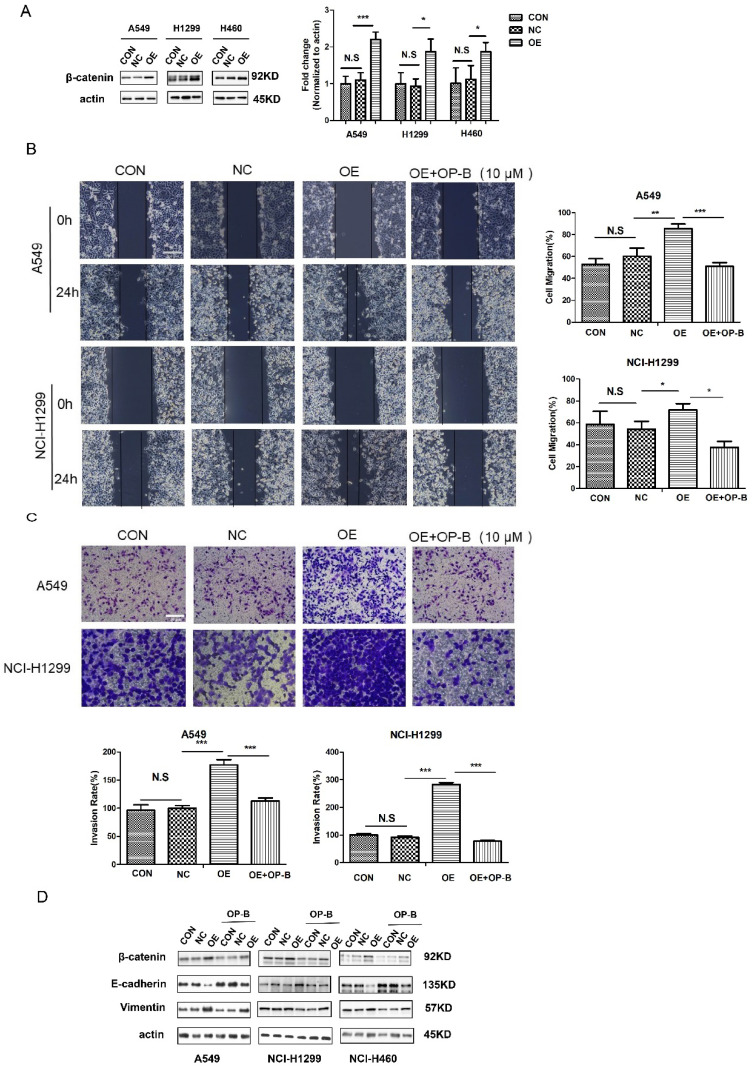
The correct new figure is shown.

**Fig 5:**
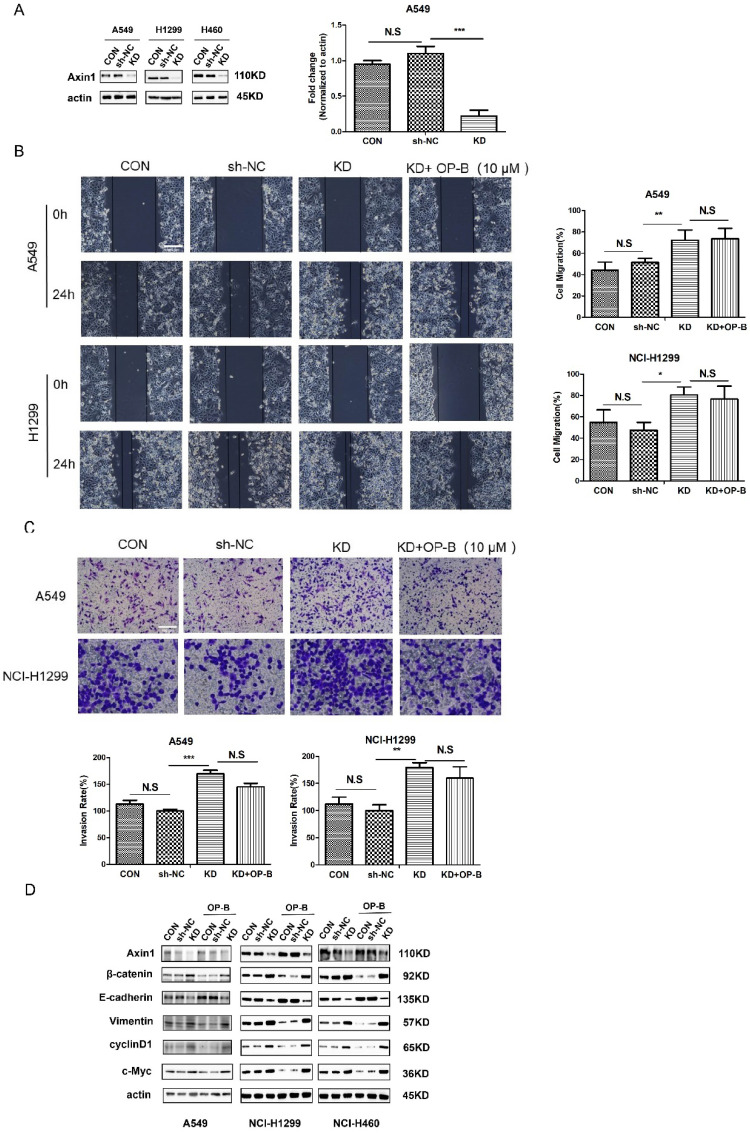
The correct new figure is shown.

